# DTEXT – text messaging intervention to improve outcomes of people with type 2 diabetes: protocol for randomised controlled trial and cost-effectiveness analysis

**DOI:** 10.1186/s12889-019-6550-6

**Published:** 2019-03-04

**Authors:** Karen Waller, Susan Furber, Adrian Bauman, Margaret Allman-Farinelli, Paul van den Dolder, Alison Hayes, Franca Facci, Lisa Franco, Alison Webb, Robert Moses, Stephen Colagiuri

**Affiliations:** 1 Illawarra Shoalhaven Local Health District, Warrawong, Australia; 2 University of Sydney, Sydney, Australia

**Keywords:** Diabetes, Text message, Mobile phone, SMS, Glycaemic control, HbA1c, Self-management

## Abstract

**Background:**

Diabetes prevalence is rapidly increasing, with type 2 diabetes predicted to be the leading contributor of non-communicable disease in Australia by 2020. It is anticipated that rates of type 2 diabetes will continue to increase if factors such as overweight and obesity, low physical activity and poor nutrition are not addressed. The majority of Australians with type 2 diabetes do not meet the guidelines for optimal diabetes management, and access to diabetes education is limited. This highlights the need for new interventions that can reduce existing barriers to diabetes education, attain greater population reach and support self-management strategies for people with type 2 diabetes.

Mobile phone text messages have shown promising results as an intervention for people with chronic disease. They have the ability to achieve high levels of engagement and broad population reach, whilst requiring minimal resources. There is however, no evidence on the effect of text messaging to improve the health of people with type 2 diabetes in Australia.

**Methods/Design:**

This randomised controlled trial aims to investigate if a 6 month text message intervention (DTEXT) can lead to improvements in glycated haemoglobin (HbA1c) and diabetes self-management among Australian residents in New South Wales (NSW) with type 2 diabetes. Community dwelling adults (*n* = 340) will be recruited with the primary outcome being change in HbA1c at 6 months. Secondary outcomes include behaviour change for diabetes self-management, self-efficacy, quality of life and intervention acceptability. An economic evaluation will be conducted using a funder plus patient perspective.

**Discussion:**

This study will provide evidence on the effectiveness and cost effectiveness of a text message intervention to reduce HbA1c and enhance self-management of type 2 diabetes in the Australian population. If successful, this intervention could be used as a model to complement and extend existing diabetes care in the Australian health care system.

**Trial Registration:**

The study has been registered with the Australian New Zealand Clinical Trials Registry, Trial ID: ACTRN12617000416392. Registered: 23 March 2017.

## Background

Type 2 diabetes is predicted to be the leading contributor to the burden of disease in Australia by 2020 [1]. In 2014–15 it was estimated that one million Australian adults (5%) had type 2 diabetes [2], and if trends continue, this figure could triple to three million by 2025, due to factors such as high rates of overweight and obesity, low physical activity and poor lifestyle behaviours [3]. The majority of Australians with type 2 diabetes do not meet the recommended guidelines for optimum diabetes management [4]. Research shows that diabetes self-management, which includes management of symptoms, treatment, physical and psychosocial consequences, and lifestyle changes [5], can improve glycaemic control and reduce complications in people with type 2 diabetes [6].

Type 2 diabetes, if not managed well, can progress to complications such as coronary artery disease, stroke, kidney failure, limb amputations and blindness [7]. These complications lead to an increased burden of the disease for the health system and the individual [8–11]. By reducing type 2 diabetes related complications, there would be a significant reduction in hospitalisations and the economic burden posed by diabetes on the health care system and people with the disease [12]. Modifiable lifestyle factors such as a poor diet and physical inactivity leading to overweight and obesity are driving the development of type 2 diabetes in the majority of cases [8]. A recent study showed that the majority of Australian adults with type 2 diabetes performed little or no exercise (66%) and had inadequate vegetable (93%) and fruit (51%) consumption [13]. The Australian Institute of Health and Welfare (AIHW) reported that only 55% of Australians with type 2 diabetes effectively manage their condition, and this figure reduces to 38% for Indigenous adults [14]. The AIHW also reported that only 18% of Australian’s complete the Annual Cycle of Care [13], the recommended best practice for the management of type 2 diabetes [7].

International studies suggest that attendance at diabetes education sessions can be as low as 30% due to logistical, medical, or financial reasons; or a perceived lack of benefit [15]. In Australia, it has been reported that over 40% of people with type 2 diabetes have no access to diabetes education programs [16], and only 24% of people saw a diabetes educator in the last year [14]. A study of Australian adults aged 18–39 years with type 2 diabetes showed that 59% chose not to participate in a structured education program with the perception that programs do not cater to their needs or concerns [17]. People not attending diabetes education have a fourfold increased risk of complications [18], therefore self-management of type 2 diabetes is critical to delaying or preventing disease progression and complications [19–21].

The use of mobile phone text message interventions to complement existing health care is a rapidly emerging field [22–24]. Text message interventions have the potential for high population reach; require minimal resources; and can be easily translated into the routine practice and existing services. In Australia, 96% of adults use a mobile phone [25], and it has been shown that usage is high amongst people from lower socioeconomic areas, rural and remote regions, the aging population and those with higher body mass index, health comprising behaviours and lower levels of self-rated health [26–29]. These socio-demographic characteristics align closely with those having a higher prevalence of type 2 diabetes [30], suggesting that a text message intervention could be an appropriate method to support self-management of their condition.

Research has shown that text message interventions can improve the short term health of cardiac patients [31], help promote smoking cessation [32] and assist in weight loss [29] by providing prompts, information, reminders and support. Studies have also shown that text messaging can aid in lowering blood pressure [33], and improve medication adherence [34], physical activity [35], weight loss [36] and glycaemic control [24, 37, 38], however, these studies have been limited due to factors such as a short study duration, small sample size and lack of theoretical basis [39–42].

A recent systematic review and meta-analysis by Faruque et al. 2017 for people with type 1 and 2 diabetes showed that text messaging interventions resulted in some improvement in glycaemic control, but not other clinical outcomes [43]. A systematic review by Dobson et al. 2017 reported that improvements in glycaemic control from text message interventions were inconclusive for people with sub optimal (HbA1c 7.1–8%) or poorly controlled diabetes (HbA1c ≥8.1%) [22]. Currently no evidence exists for a text message intervention to improve the health and support self-management of adults with type 2 diabetes in Australia.

## Aim

This study aims to determine the effectiveness and cost effectiveness of a text message intervention (DTEXT) on improvements in glycated haemoglobin (HbA1c) and self-management for people with type 2 diabetes.

## Methods

### Design

**Fig. 1:**
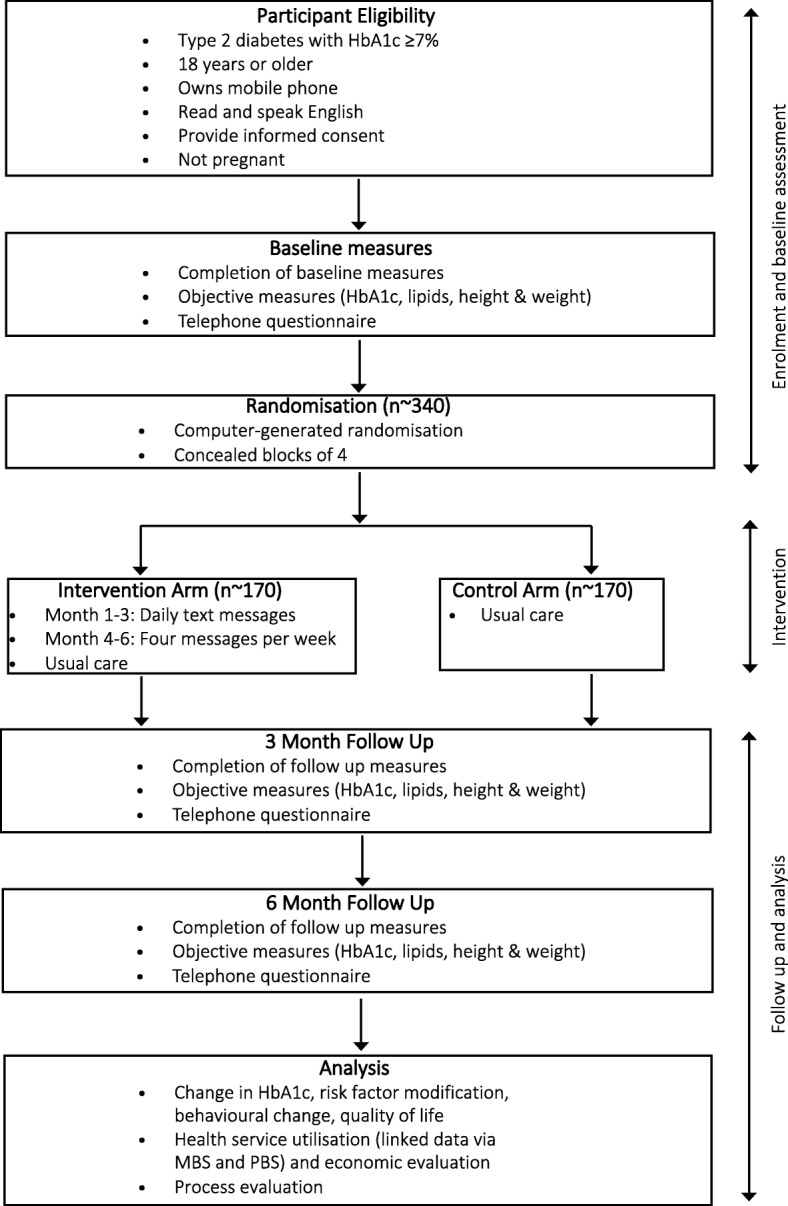
Study design and flow. HbA1c: glycated haemoglobin, MBS: Medicare Benefits Schedule, PBS: Pharmaceutical Benefits Scheme

This study protocol describes a 6-month text message intervention (DTEXT) that will be conducted as a randomised controlled trial, with two parallel arms, an intervention arm and a control arm - see Fig. 1.

The study protocol is in accordance with the SPIRIT 2013 statement [44], the Consolidated Standards of Reporting (CONSORT) – eHEALTH checklist [45] and Hoffman et al’s 2014 [46] extension of these two documents.

### Participants

Participants will be community dwelling adults with a diagnosis of type 2 diabetes and an HbA1c of 7% (53 mmol/mol) or above, who reside in New South Wales, Australia. Participants must be 18 years or older, own and be able to use a mobile phone, be able to read and speak English, and be able to provide informed consent. Exclusion criterion is pregnancy, to exclude gestational diabetes.

### Recruitment

Participants will be recruited by referral from health service providers and doctors; advertising in newspapers, community noticeboards, radio and social media (Facebook); and mail outs through the regional public health Diabetes and Renal Services in the Illawarra Shoalhaven Local Health District, and the Australian Government National Diabetes Services Scheme.

### Sample size

The required sample size of 340 (170 per arm) will provide 80% power of detecting a between-group change in HbA1c of 0.7%, allowing for 20% loss to follow up and an alpha = 0.05.

### Randomisation and blinding

The PROC PLAN function in Statistical Analysis System (SAS) v 9.4 will be used to randomise participants into sets of two letters (A and B, representing the intervention and control arms respectively) by blocks of four to ensure a balanced sample size across both study groups. Couples will be treated as a single unit to ensure both are allocated to the same treatment group.

The randomisation letters will be generated by a statistician at the University of Sydney, and packaged by a NSW Health staff member not involved in the study to ensure group allocation is concealed from the researcher until completion of baseline measures.

Participants will be randomised into the intervention or control arm after providing informed consent, obtaining medical clearance and completing baseline measures. Due to the nature of the intervention and telephone surveys conducted, participants and researchers cannot be blinded to treatment allocation. Pathology collectors assessing objective measures (HbA1c, lipids, height and weight) and the participant’s doctor will not be informed of treatment allocation.

## The intervention

The randomised controlled trial will involve two parallel arms, an intervention arm and a control arm. The intervention arm will receive the 6 month text message intervention (DTEXT). Both arms will receive usual care from their treating doctor and associated health professionals.

The text message intervention was developed by an expert panel of clinicians, academics and health promotion staff using evidence-based guidelines and recommendations [7], and the use of appropriate language for Australian adults with type 2 diabetes [47, 48]. The content of the messages has a readability level of grade 5–6 (10–11 years of age) [49]. Each text message was limited to 160 characters in length, and no message was repeated during the intervention. The messages were tested with 20 people with type 2 diabetes in a 5 week pilot program to determine message acceptability and ensure message content, tone and structure was appropriate. Adjustments to the messages were made based on pilot participant feedback.

The DTEXT text messages were designed using Michie et al’s COM-B system from the Behaviour Change Wheel [50]. This system increases the individual’s capability, opportunity and motivation to enhance their self-management of health and lifestyle related behaviours, with the aim to improving diabetes control. Individual behaviour change is a critical component to achieve self-management of conditions [20]. The behaviour change techniques used in the text messages were based on the Behaviour Change Technique Taxonomy [51] and include: providing information about the health consequences, prompts and cues, problem solving, self-monitoring of behaviour and outcome, goal setting of behaviour and outcome, instruction on how to perform a behaviour, graded tasks, social support, social comparison, self-talk, credible source and reducing negative emotions. Behaviour change techniques are recommended for disease management programs using mobile technology [52].

The text message intervention will consist of daily text messages for months 1–3; and four messages per week for months 4–6. The messages will be designed to be sent at random times between 8 am and 5 pm, with message themes coinciding with relevant times of the day e.g. breakfast messages sent in the early morning, healthy dinner options sent in the afternoon. They include modules on nutrition, physical activity and sedentary behaviour, diabetes care and compliance with the diabetes Annual Cycle of Care, weight management, medication adherence and smoking cessation. The messages will be semi-personalised with occasional use of the participant’s name receiving the messages, occasional use of the researcher’s name (Karen) sending the messages, and tailored so that only smokers receive smoking cessation messages and only those taking medication will receive medication adherence messages (see Table 1).

**Table 1:** Summary of the 6 month DTEXT intervention

Module	Description	Number of Messages n (%)	Example message
Nutrition	Up to 3 messages a week on dietary advice and recommendations; eating out; portion size; example meal, snack and drink options.	47 (35)	For good health, aim to eat 5 serves of vegetables each day. 1 serve = half a cup cooked vegetables or 1 cup salad vegetables. Try eating some with each meal.
Physical activity	Up to 3 messages a week on physical activity advice and recommendations; reducing sedentary time; goal setting; free or home based activities; community programs and support.	47 (35)	Hi (name), Did you know by walking up 2 flights of stairs per day you can lose 3 kg a year? Are there any stairs nearby you could try? Karen
Diabetes care	Up to 2 messages per week on: self-management; completing the Annual Cycle of Care; and accessing health professionals for care and support.	26 (20)	HbA1c tests are the best measure of your diabetes control. Aim to keep HbA1c under 7% or 53 mmol and have it tested each year. Ask your doctor about your HbA1c.
Weight management	Up to 1 message per week with motivation and guidance to achieve and maintain a healthy weight range.	13 (10)	Sugary drinks (soft drink, sports drink, cordial) can lead to weight gain – a 600 ml soft drink bottle has 16 teaspoons of sugar!
Medication adherence	Up to 1 message per fortnight with support for medication adherence; filling scripts; seeking advice for adverse effects; and undertaking an annual medication review.	9 (additional messages for medication users)	Have you taken your diabetes medication today? Try using a calendar reminder or ask a pharmacist about a weekly pill box to make taking your medication easier.
Smoking cessation	Up to 1 message per fortnight with advice, motivation and support for quitting smoking; and accessing community support programs.	9 (additional messages for smokers)	For help quitting smoking call the Quitline on 137848. Quitline staff are trained to help you manage your journey to better health.

A technology platform to deliver text messages will be developed using the software company Message Media. The platform will allow for automation of the text message intervention. The intervention will be designed as a uni-directional program, however participants will be able to reply to text messages with responses addressed by the study team, if required. Participants can withdraw from the study at any time using an automated opt-out STOP function.

### Outcomes measures

#### Primary outcome

The primary outcome measure will be change in HbA1c, determined by blood test at baseline, 3 and 6 months. Blood tests will occur at the participant’s local pathology collection centre, and a copy of the results will be sent to their doctor for consultation and follow up with their patient if required. Participants will receive a $25 store voucher upon receipt of pathology results.

#### Secondary outcomes

Secondary outcome measures will be collected at baseline, 3 and 6 months through objective measures taken by pathology collectors and a self-report telephone questionnaire administered by the research team. The questionnaire was piloted with 10 people and determined to be acceptable by pilot participants and easy to deliver with an appropriate timeframe (10–15 min) by the research team.

#### Secondary outcome measures include

##### Blood lipid profile

This includes total cholesterol (TC), low density lipoprotein (LDL-C), high density lipoprotein (HDL-C) and triglycerides (TG). Lipid results will be assessed according to the recommended guideline levels for people with type 2 diabetes [7].

##### Body mass index (BMI)

Weight and height will be assessed for calculation of BMI (kg/m^2^). Readings will be recorded by standardised pathology collectors at the time of each blood test and through self-reported data collected during the questionnaires. BMI status will be assessed according to the World Health Organisation classifications [53].

##### Physical activity

This will be measured using the validated two-question (2Q) assessment tool. This captures duration, frequency and intensity of activity to determine if people are meeting the current guidelines for physical activity [54].

##### Nutrition

This will be measured using selected questions from the New South Wales Population Health Survey [55] and a question developed specifically for this study. The survey provides detailed information on the health of adults in NSW for planning and evaluation purposes.

##### Smoking status

This will be measured using a question from the World Health Organisation (WHO) Steps Instrument and a question developed specifically for this study. The Steps instrument is used to collect and measure non-communicable disease risk factor data [56].

##### Quality of life

This will be assessed using the Short Form 12-item Health Survey (SF12v2). This multi-purpose survey measures quality of life in terms of functional health and wellbeing and has two subscales addressing psychometric measures of physical and mental health [57]. Preference-based health utilities will be determined from a subset of the SF12 questions, using the UK valuation algorithm [58]. These utility scores will be used to calculate Quality-adjusted Life Years (QALYs) for the economic evaluation.

##### Self-efficacy (physical activity, nutrition and diabetes self-care)

This will be measured by questions developed specifically for this study to determine confidence levels with activities.

##### Medication adherence

This will be measured using a question developed specifically for this study to determine compliance with medication schedules.

### Statistical analysis

Statistical analysis will be conducted using the software programs Statistical Package for the Social Sciences (SPSS-25) and Statistical Analysis System (SAS) v9.4, with an intention to treat principle.

Generalised estimating equation (GEE) models will be used to assess impact, modelling repeated outcome measures over time. Change in HbA1c will be analysed using GEE models to test the effect of the intervention on change in HbA1c between the two groups.

GEE will also be used to assess the effect of group allocation on changes over time in BMI, TC, LDL-C, HDL-C, TG, physical activity, dietary behaviour, smoking status, quality of life, self-efficacy measures and use of health services after adjusting for baseline scores. Two planned subgroup analyses will be undertaken to assess differential impact of the intervention on the basis of age and sex.

### Economic evaluation

The economic evaluation will be conducted from a health funder plus patient perspective. The ‘within trial’ economic evaluation will include a cost-effectiveness and a cost-utility analysis.

The cost of delivering the intervention, including setting up the technology platform, delivery of text messages, staffing costs and consumables, will be determined using standard micro-costing techniques. Healthcare utilisation, including doctor or specialists visits, will be determined by individual data linkage to the Medicare Benefits Schedule. Medicines use will be determined from the Pharmaceutical Benefits Schedule. Individual patient consent will be obtained to access records from these administrative databases. The records will provide both health funder (government) cost and patient out-of-pocket costs. All costs will be valued in 2018 Australian dollars.

The main outcome for the cost-effectiveness analysis will be the incremental cost of the intervention per unit of HbA1c avoided compared with usual care. The cost-utility analysis will estimate the incremental cost per quality adjusted life year (QALY) gained. Quality of life will be measured using the SF12v2 at baseline and end of trial and quality adjusted life years calculated using the United Kingdom valuation [58].

Using the mean costs and mean health outcomes in each trial arm, incremental costs and incremental outcomes will be determined. These results will be plotted on a cost-effectiveness plane with bootstrapped estimates to present joint uncertainty in costs and health outcomes. Multiple one-way sensitivity analyses will be conducted around key variables. A cost-effectiveness acceptability curve (CEAC) will be derived which estimates the probability of the intervention being cost-effective at different willingness to pay thresholds.

### Process evaluation

Process evaluation will be conducted using survey responses and process information obtained in the telephone questionnaire by interview at 3 and 6 months. The evaluation will assess intervention acceptability based on participant satisfaction, engagement, retention and recommendations. These measures will be summarised using descriptive statistics.

## Discussion

The study will examine if the text message intervention, DTEXT, leads to improved glycated haemoglobin and self-management of type 2 diabetes compared to usual care. If successful, DTEXT will provide evidence to support scalable text message interventions as an appropriate tool to complement existing health care services and assist in improving the health of Australians with type 2 diabetes.

The findings from DTEXT have the potential to inform the New South Wales Ministry of Health on the scalability and translation of the intervention across the health care system. If the intervention is effective it could provide benefits on a population, individual and health service delivery level. These benefits could include: improving the population reach of diabetes education and support through an intervention that is equitable across location, culture and socioeconomic status; enhances diabetes self-management; and reduces diabetes related complications and hospitalisations with a low-cost, low resource intensive intervention that can enhance existing models of care.

## Abbreviations

AIHW: Australian Institute of Health and Welfare
BMI: Body mass index
CEAC: Cost effectiveness acceptability curve
COM-B system: Capability opportunity motivation behaviour system
CONSORT: Consolidated standards of reporting
DTEXT: The name of the diabetes text messaging intervention
GEE: Generalised estimating equations
HbA1c: Glycated haemoglobin
HDL-C: High density lipoprotein cholesterol
LDL-C: Low density lipoprotein cholesterol
MBS: Medicare benefits schedule
NSW: New South Wales
PBS: Pharmaceutical benefits scheme
QALYS: Quality-adjusted Life Years
SAS: Statistical analysis system
SF12v2: Short Form 12 item Health Survey, version 2
SMS: Short Messaging Service
SPSS-25: Statistical Package for the Social Sciences
TC: Total cholesterol
TG: Triglycerides
WHO: World Health Organisation
